# Large-Scale, Wavelet-Based Analysis of Lysosomal Trajectories and Co-Movements of Lysosomes with Nanoparticle Cargos

**DOI:** 10.3390/cells11020270

**Published:** 2022-01-13

**Authors:** Konstantin Polev, Diana V. Kolygina, Kristiana Kandere-Grzybowska, Bartosz A. Grzybowski

**Affiliations:** 1 Center for Soft and Living Matter, Institute for Basic Science (IBS), Ulsan 44919, Korea; conspol7@gmail.com (K.P.); kolyginadv@gmail.com (D.V.K.); 2 Department of Biomedical Engineering, Ulsan National Institute of Science and Technology (UNIST), 50 UNIST-gil, Ulsan 44919, Korea; 3 Department of Chemistry, Ulsan National Institute of Science and Technology (UNIST), 50 UNIST-gil, Ulsan 44919, Korea

**Keywords:** lysosome transport, cancer lysosomes, mixed-charge nanoparticles, lysosome-nanoparticle co-movement, confocal reflection microscopy, continuous wavelet transform, maximum-likelihood estimates, lognormal distribution

## Abstract

Lysosomes—that is, acidic organelles known for degradation/recycling—move through the cytoplasm alternating between bursts of active transport and short, diffusive motions or even pauses. While their mobility is essential for lysosomes’ fusogenic and non-fusogenic interactions with target organelles, their movements have not been characterized in adequate detail. Here, large-scale statistical analysis of lysosomal movement trajectories reveals that lysosome trajectories in all examined cell types—both cancer and noncancerous ones—are superdiffusive and characterized by heavy-tailed distributions of run and flight lengths. Consideration of Akaike weights for various potential models (lognormal, power law, truncated power law, stretched exponential, and exponential) indicates that the experimental data are best described by the lognormal distribution, which, in turn, can be related to one of the space-search strategies particularly effective when “thorough” search needs to balance search for rare target(s) (organelles). In addition, automated, wavelet-based analysis allows for co-tracking the motions of lysosomes and the cargos they carry—particularly the nanoparticle aggregates known to cause selective lysosome disruption in cancerous cells. The methods we describe here could help study nanoparticle assemblies, viruses, and other objects transported inside various vesicle types, as well as coordinated movements of organelles/particles in the cytoplasm. Custom-written code that includes integrated workflow for our analyses is made available for academic use.

## 1. Introduction

Lysosomes are acidic (pH ~4.2– 5.5), membrane-bound terminal compartments for degradation and recycling of cargos delivered to them through endocytosis and autophagy [1]. Lysosomes also function as docking sites for signaling complexes [2,3], engage in physical contacts with other organelles [2,4], and repair plasma membrane through lysosomal exocytosis [5,6]. Malignant transformation results in alterations in the function and composition of lysosomes—cancer lysosomes are larger [7], and with few exceptions, have lower pH [8], increased proteolytic activity, altered membrane composition and spatial distribution, and display enhanced secretion of their contents, notably protons, H^+^, and proteases, towards extracellular space [9,10]. Since the resulting acidification and proteolytic remodeling of the tumor microenvironment drives invasion, there is hope that inhibition of excessive lysosomal exocytosis could limit cancer metastasis [11,12].

To perform these diverse functions, both cancer and healthy cells’ lysosomes rely on their vigorous movements throughout the cytoplasm [1,2]. As for most organelles, lysosomes move in a “stop-and-go” manner where periods of fast, directional runs alternate with short, random movements and pauses [13,14]. The rapid, long-range directional “runs/flights” are due to active, ATP-dependent transport of lysosomes by kinesin (towards the periphery) and dynein (towards cell center) motors along the microtubule (MT) tracks [15]. The short movements and pauses are due to constraints imposed by MT intersection points [16], other organelles [4], lysosome interactions with actin-based motors [17], or their transport along a subset of detyrosinated MTs [18]. These movements are often characterized by mean square displacement (MSD), which has time dependence $t^{\alpha }$ [17,19,20], where exponent α indicates the type of movement: α<1 subdiffusive, constrained movement; α≅1 passive transport by pure diffusion; α≥2 ballistic/directional movement; and 1<α<2 superdiffusive movements observed in complex environments and sometimes indicating Lévy walks. Moreover, the detailed character of movement and, in some cases, the nature of the underlying mechanisms can be inferred by studying the distributions of persistent motions (runs and flights) using maximum likelihood estimates (MLE) to fit experimental data and comparing multiple competing models [21,22,23,24,25]. Even though the intermittent nature of lysosomal transport is easy to recognize visually, the proper assignment of transition points between active and diffusive trajectory segments, and thus the construction of run/flight statistics (expressed as the so-called cumulative distribution functions, CDFs), is not trivial. Such assignments are performed either manually [26], or by speed correlation index [27], trajectory asymmetry [28], temporal MSD analysis [18,29,30,31], multimodal and “self-similar” sub-trajectory analysis [32,33], Hurst exponent estimation [34], or with wavelet analysis [35,36]. The latter approach is a particularly excellent choice because it is highly effective at detecting transient heterogeneous changes in dynamics embedded in particle trajectory data. For example, Granick’s team applied a wavelet-based approach to show that exponentially distributed “runs” of EGF (or LDL)-containing endosomes self-organize into “flights” that are drawn from a truncated power-law distribution, thus consistent with Lévy walks [24]. While intriguing, these studies have not yet been extended to other vesicle types, and patterns of lysosome movements have not been previously studied in such detail. In addition, one outstanding question concerns the movement characteristics of lysosomes in cancer versus healthy, normal cells. It remains unclear if cancer-associated lysosome alterations (cf. above) also lead to a change in lysosome movement patterns.

In this context, we [37,38] and others [39,40] have exploited the differences between normal and cancer cells’ lysosomes (cf. above) to target the latter selectively. In particular, our own strategy has been to target the lysosomes with gold nanoparticles, AuNPs, that are engineered to precipitate in regions of specific pH. We showed that such NPs coated with mixtures of positively and negatively charged ligands at precisely defined proportions, so-called mixed-charge nanoparticles (MCNPs) [41,42], assemble into micron-size crystals inside the low-pH cancer lysosomes, ultimately inducing selective cancer cell death [37,38]. The aggregation of the same nanoparticles is limited in noncancerous cells; instead, they accumulate in higher-pH autolysosomes and are cleared via exocytosis. Unlike cationic amphiphilic drugs (CADs) [40], MCNPs do not induce robust permeabilization of cancer lysosome membranes but rather increase cancer lysosome sizes, displace signaling proteins from their membranes, and sensitize them to damage [37].

In the course of this work, we performed multiple high-resolution imaging studies tracking the trajectories of individual lysosomes in normal vs. cancer cells, both in the absence and in the presence of MCNPs cargos [37]. In the end, we assembled a uniquely diverse and large collection of 60,431 lysosomal trajectories coming from five cell types (sampled every 200 ms for 3–5 min), and containing 67,764 periods of active transport. Qualitative analysis suggested disrupted cancer lysosome transport by MCNPs, but was not accompanied by any quantitative analyses of pathway structure [37]. Here, we perform such analyses with two overriding objectives in mind: (1) to perform a rigorous statistical comparison of movement patterns of untreated cancer and normal cells’ lysosomes; and (2) to quantitatively assess the effect of MCNP cargos on lysosome transport in cancer versus normal cells. To address the latter question, we developed a method based on continuous wavelet transform (CWT) for detecting particles/vesicles that undergo conjoint movement; to address the former, we implemented the detection of active motion that is also based on CWT [35]. The results of these analyses not only confirm the previously established superdiffusive nature of lysosomal motions in both normal and cancer cells, but also indicate that these motions are typically characterized by lognormal distribution of run/flight lengths, which may point to the efficiency in which lysosomes “patrol” the cytoplasm to find target organelles, such as endosomes, phagosomes, and autophagosomes. Of note, the appropriateness of the lognormal fit is corroborated by the evaluation of other possible models (i.e., power law, truncated power law, stretched exponential, and exponential). The methods we describe here can be extended to study in-cell motions of other types of synthetic particles or viruses [43], as well as coordinated organelle movements [4] or “cargo” transport by hitchhiking onto other motor-driven organelles [44,45,46]. Such analyses will be facilitated by the house-written code for our integrated workflow—this code is made available to academic users at https://github.com/conspol/cwt-active-comovement, accessed on 10 January 2022.

## 2. Materials and Methods

### 2.1. Cell Culture, Nanoparticle Treatments, and Confocal Microscopy

The cell culture, confocal imaging, and mixed-charge nanoparticle (NP) synthesis were previously described in [37]. Briefly, mouse embryonic fibroblasts (MEF) were a gift from X. Tong (Northwestern University Medical School, Chicago, IL, USA). Human fibrosarcoma HT1080 (CCL-121), human breast epithelial cells MCF-10A (CRL-10317), and human breast adenocarcinomas MDA-MB-231 (HTB-26) and MCF7 (HTB-22) were purchased from American Type Culture Collection (ATCC). MEF and HT1080 cells were cultured in Dulbecco’s modified Eagle medium (DMEM, 11995-065, Thermo Fisher Scientific, Waltham, MA, USA) with 10% fetal bovine serum (FBS, TMS013BKR, Merck Millipore, Burlington, MA, USA) and 25 μg mL^−1^ gentamycin. MCF-10A cells were cultured in DMEM/F12 (11330-032, Thermo Fisher Scientific, Waltham, MA, USA) with 5% horse serum, 20 ng mL^−1^ epidermal growth factor, 10 µg mL^−1^ insulin, 0.5 mg mL^−1^ hydrocortisone, 100 ng mL^−1^ cholera toxin, and penicillin/streptomycin (10,000 U mL^−1^ penicillin and 10,000 µg mL^−1^ streptomycin). MCF-7 were cultured in RPMI with 10% FBS and 25 μg mL^−1^ gentamycin. MCF-10A, MCF-7, HT-1080 and MEF cells were cultured in a 5% CO_2_ atmosphere at 37 °C. MDA-MB-231 were cultured in L-15 medium (21083027, Thermo Fisher Scientific, Waltham, MA, USA) with 10% FBS and 25 μg mL^−1^ gentamycin at 37 °C without CO_2_. All cell lines used in this study were free of mycoplasma.

NP synthesis and characterization were described in references [37,47]. Briefly, NPs with Au cores of average diameter *d* ≈ 5.3 nm (from TEM) were coated with mixtures of negatively charged 11-mercaptoundecanoic acid (MUA, 450561, Merck Sigma-Aldrich, Burlington, MA, USA) and positively charged N,N,N-trimethyl(11-mercaptoundecyl)ammonium chloride (TMA, FT#006 ProChimia Surfaces, Gdansk, Poland) ligands. Out of several ligand-layer compositions, the “80:20” particles caused the death of cancer cells most selectively—these mixed-charge nanoparticles (MCNPs) had the on-particle ratio of TMA to MUA ligands of 80:20 [41] and zeta potential, ζ ~ +21.4 ± 3.6 mV [37]. Accordingly, most of the analyses described here are based on the “80:20” MCNPs, although, as controls, purely cationic NPs coated with only TMA ligands were also considered; these NPs had ζ ~ +27.5 ± 5.1 mV. The hydrodynamic diameters of all ligand-stabilized particles were D_H_ ≈ 7.8 nm (from DLS).

For imaging, cells were cultured on glass-bottom cell culture dishes (P35G-1.5-20-C, MatTek, Ashland, MA, USA) coated with fibronectin (25 µg mL^−1^). Subconfluent cells were then cultured without NPs (Control/no-NPs), or continuously exposed to 80:20 or TMA NPs (50 nM) for 8 h (for HT-1080 and MEF), or 24 h (for MCF-10A, MDA-MB-231 and MCF-7) at 37 °C. The NP concentrations/exposure times were chosen to allow NP aggregation inside cells’ lysosomes but not induce cell death during lysosome tracking experiments. Overall, at higher concentrations and/or longer exposure times, TMA NPs are toxic, while 80:20 NPs are selectively cytotoxic only towards cancer cells. A detailed description of the cell responses is provided in [37]. Both cancer and noncancerous cells readily internalized both types of nanoparticles, though 80:20 nanoparticles aggregated more readily in lysosomes than TMAs. NP clusters—larger in cancer cells and smaller in noncancerous cells—were imaged label-free with confocal reflection microscopy as described previously [37,47].

Acidic lysosomal organelles were labeled with 50 nM LysoTracker Red DND-99 (L7528, ThermoFisher Scientific, Waltham, MA, USA) for the last 30 min of NP exposure time. Live cells were imaged immediately with a Nikon A1R confocal microscope using ×60, 1.4 NA (numerical aperture) or ×100, 1.45 NA oil immersion objectives. The confocal reflection mode for observing aggregates of Au NPs was set up to use the 638 nm laser, and the reflection/scattering signals were collected at 663–738 nm. LysoTracker Red DND-99 was excited using a 561 nm laser line, and the emission was collected at 560–618 nm. Collecting a wide range of emissions for the Reflection channel was due to the constraints of the available filters/configuration options with standard PMT detectors of the Nikon A1R microscope. Time-series images of lysosomes and Au NP aggregates were collected with resonant scanner mode at time-steps/frames ~200 ms (5.11 fps) for total time ~3–5 min yielding movies with ~900–1540 time-steps/frames. All cells were imaged in their complete cell culture medium with the temperature, and CO_2_ concentrations maintained using a stage-fitted incubator and gas mixer (Live Cell Instruments, Seoul, Korea) as described previously [37,47].

### 2.2. Image Processing and Tracking

All image post-processing and particle tracking steps were performed with NIS-Elements AR (Nikon, Tokyo, Japan) software v4.50 or v5.02. To reduce the noise from images acquired with a resonant scanner, the rolling average (using menu ND Image Average) was computed over five consecutive images. Particle detection and tracking were performed using NIS-Elements’ particle tracking module. Gaps of a maximum of 3 frames were allowed. The trajectories that spanned less than 20 frames (~4 s) were excluded from the analysis. Trajectories were exported and analyzed with a house-written code (cf. next section).

### 2.3. Movement Analysis

#### 2.3.1. Continuous Wavelet Transform

Coordinates of each axis (*x* and *y*) of all particles’ (lysosomes or NP aggregates) trajectories were processed via continuous wavelet transform (CWT):

$$C\left(t_{0},a\right)=\frac{1}{a}\int_{\mathbb{R}}s\left(t\right)\psi (\frac{t-t_{0}}{a})dt$$

where $s\left(t\right)$ is a time series (in our case, coordinates over time), and ψ is a wavelet function having scale (width) a, centered at time $t_{0}$. We used the Haar wavelet, which is a “square-shaped” function of a form:

$$\psi \left(t\right)=\left\{\begin{array}{c} 1,\;0\leq t<1/2\\ -1,\;\frac{1}{2}\leq t<1\\ \;0,\;\mathrm{otherwise} \end{array}\right.$$

**Figure 1:**
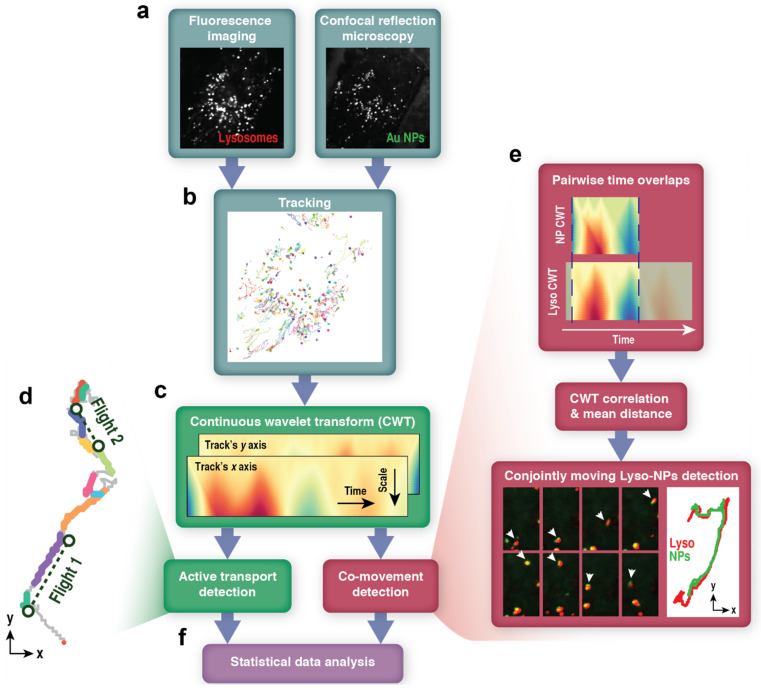
Schematic of the analysis of lysosome movement patterns and lysosome-nanoparticle co-transport. (**a**) A snapshot of a time-series of an MCF-10A cell with lysosomes marked by Lysotracker Red and imaged with fluorescence and gold nanoparticle aggregates (Au NPs) imaged label-free in confocal reflection mode (CRM). See also Supplementary Movies S1 and S2. (**b**) Both lysosomes and Au NP aggregates are tracked by a single-particle tracking module in NIS-Elements software. (**c**) Multiscale wavelet coefficients are computed by continuous wavelet transform (CWT) for x and y coordinates of trajectories. (**d**) Shows an example of lysosome trajectory segmented using CWT-based active transport detection. The color indicates intervals of diffusive motion (grey) and directional, active transport—several “runs” (colored) that often join to form directional “flights” (dashed lines connect the beginnings with ends of such flights). (**e**) CWT results are also used to detect co-transported lysosomes and Au NP aggregates. (**f**) Classifications performed by the previous stages of the workflow are used in the statistical data analysis, investigating lysosome mean square displacements (MSD) and persistence lengths for runs (*l*) and flights (*L*). See the Section 2 for more details.

Using scales 1 to 50, we obtained 50-by-t “maps” of wavelet coefficients $C\left(t,\;a\right)$ (for examples, refer to Figure 1c). The coefficients $C\left(t,\;a\right)$ were then used in two types of analysis: extraction of periods of active transport by lysosomes and detection of conjoint movement of lysosomes with NP aggregates. For more information about the wavelet transform, please see Discussion and references therein.

#### 2.3.2. Active Transport Detection

CWT-based active transport detection for intracellular vesicles is described in [35]. We followed the method without modifications and performed parameter tuning as prescribed by the authors. Briefly, after surveying the wavelet transform coefficients for lysosomal trajectories, we set the initial wavelet scale $\tilde{a}$ such that the effects of the smallest movements and noise are reduced, but the information was not lost due to the merging of bands. First and last $\tilde{a}/2$ time points were removed since the movement information at these times was not represented correctly after CWT due to edge effects. We then applied a universal threshold, projected from the wavelet scale a=2, calculated as:

$$\delta =r\sigma_{2}\sqrt{2\ln N\tilde{a}/2}$$

where N is the number of data points in the trajectory (excluding $\tilde{a}$ time steps), r is the pre-factor (initially set to 1) and $\sigma_{2}$ is an estimate of the standard deviation of noise on the scale of 2:

$$\sigma_{2}=\frac{median_{i}\left(\left|C\left(i,\;2\right)\right|\right)}{0.6745}$$

where $C\left(i,2\right)$ is the wavelet coefficient at the time point i of scale 2. The time points with scale $\tilde{a}$ wavelet coefficients $C\left(i,\tilde{a}\right)$ above the threshold were classified as active “runs.” After the calculations, we visually inspected the result of classification for the chosen $\tilde{a}$ and compared the assignment to the trajectories’ mean square displacements (MSD). The choice of $\tilde{a}$ was not sensitive to small changes, and the outcome of classification was on the conservative (having false-negative errors) side, as expected [35]. We then decreased the pre-factor r until the amount of false negative classifications diminished. For our data, with $\tilde{a}=20$ and r=0.8 the amount of false negative errors was minimized, while false positives errors were not found by visual inspection.

To determine persistent “flights,” we followed the error-radius method described elsewhere [24,48]. In brief, runs were merged into flights if the run’s coordinate points lay within some “corridor” whose width adapted to the given trajectory. The corridor width equal to 1.27 of the run’s maximum width (perpendicular to the line connecting start- and end-points of the run), but not smaller than 0.4 μm, was chosen for our data by minimizing the autocorrelation of turning angles between the resulting flights. Additionally, to ensure that very short runs are not erroneously merged with runs having a very wide spread (i.e., with large corridor width), runs with turning angles (between the start-stop lines of consequent runs) larger than 120° were filtered out.

**Figure 2:**
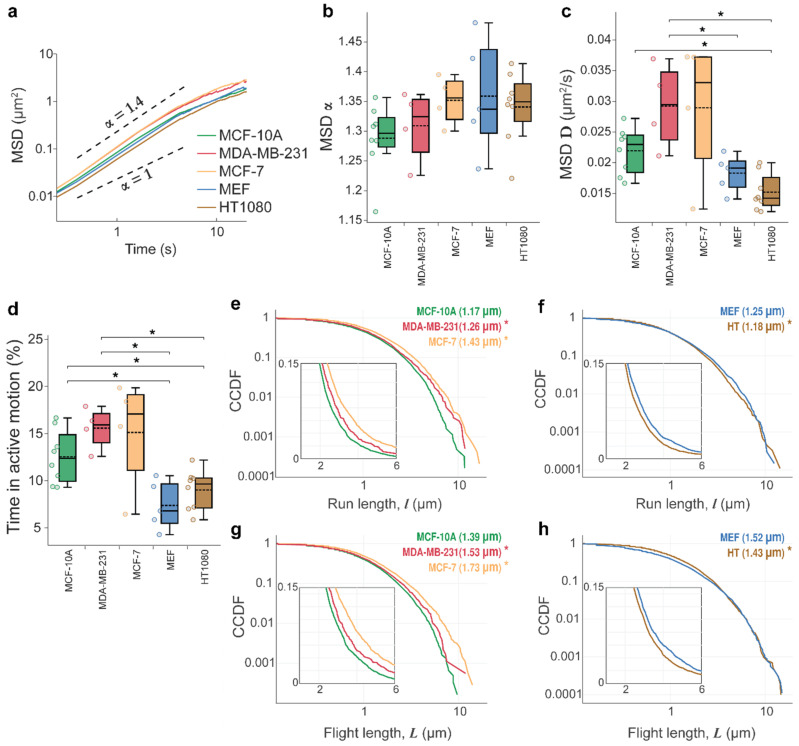
Lysosome movements are superdiffusive and fit the lognormal distribution. (**a**) Log-log plots of the lysosomes’ mean square displacements (MSD) versus time, MSD ∝ *t*^α^, with all trajectories for all cells from one type pooled together where α > 1 indicates superdiffusive lysosome movements for all cell types. See Supplementary Movie S1. Box-plots showing (**b**) exponent α and (**c**) diffusion coefficient, *D*, and (**d**) % time spent in active motion. Data are displayed as box-and-whisker plots; boxes delineate the lower and upper quartiles of the data, middle lines show median values, dashed lines show mean values for each cell type, colored dots are data points for each analyzed cell, and whiskers show upper and lower extremes. For (**b**–**d**), all trajectories in each analyzed cell were pooled together, and mean values for each cell were computed. The latter are shown as data points in the box plots. MCF-10A (*n* = 8 cells, *l* = 5260 lysosome trajectories), MDA-MB-231 (*n* = 4, *l* = 1076); MCF-7 (*n* = 4, *l* = 1756), MEF (*n* = 5, *l* = 5781), and HT-1080 (*n* = 8, *l* = 7953). (**e**–**h**) The complementary cumulative distribution functions, CCDFs, for run and flight lengths detected with wavelet analysis and plotted on a log-log scale; insets highlight the difference on a linear scale, which otherwise is not as apparent in this region of the logarithmically scaled plots. Noncancerous breast epithelial MCF-10A cell line is compared against MDA-MB-231 and MCF-7 breast adenocarcinomas. Mouse embryonic fibroblasts (MEF) are compared against the HT-1080 fibrosarcoma cell line. Asterisk denotes statistically significant differences between run/flight lengths for cancer cells compared with noncancerous counterparts determined by a Cramer–von Mises criterion (* *p* < 0.01). Only the significant differences are shown. The number of cells and runs/flights analyzed were as follows: MCF-10A (r = 5742 runs, f = 4830 flights), MDA-MB-231 (r = 1683, f = 1384), MCF-7 (r = 3410, f = 2829), MEF (r = 6156, f = 5060), and HT-1080 (r = 8697, f = 7189). The statistical parameters are shown in Table 1, and model fits are shown in Figure A1.

Percent of time spent in active transport (for Figure 2d) was computed as a ratio of time while lysosomes performed “runs” vs. total time of tracks with first and last $\tilde{a}/2$ points of each track excluded to avoid bias (since active motion detection algorithm did not consider those points).

The code for performing these analyses is available as a part of our workflow posted at https://github.com/conspol/cwt-active-comovement, accessed on 10 January 2022.

#### 2.3.3. Co-Movement Detection

We first identified the time overlaps for every lysosome-NP aggregate pair in the cell. Then, we isolated the CWT coefficients corresponding to the overlap time for each pair that had the overlap, thus obtaining a pair of same-sized matrices. For each pair (one pair for each axis, *x* and *y*), we calculated Pearson’s correlation coefficient:

$$P_{j}=\frac{cov\left(X_{wav},\;Y_{wav}\right)}{\sigma_{X_{wav}}\sigma_{Y_{wav}}}$$

where j is trajectories’ axis (x or y), $X_{wav}$ and $Y_{wav}$ are wavelet time and scale axes. We considered the movements correlated if both $P_{x}$ and $P_{y}$ were larger than 0.7. The lysosome–NPs pair was considered co-moving if the movements of the two particles were correlated, and the average of their center-to-center distance over time was below 1 μm.

### 2.4. Statistical Data Analysis

The data were statistically analyzed using Python code (also available at https://github.com/conspol/cwt-active-comovement, accessed on 10 January 2022). Fits were performed using routines from SciPy [49] and “power law” [50] packages. MSD curves were fit in the time range from 0 to 4 s (where lines are mostly straight; effects of a limited cellular space start to manifest on longer timescales, evident by downward regions on MSD curves in Figure 2a). The parameters for power law and exponential distribution fits were obtained using analytical expressions for corresponding maximum likelihood estimators [21]; for lognormal, stretched exponential, and exponentially truncated power law distributions, numerical maximum likelihood estimation was used. Equations of the distributions’ probability density functions are in Appendix B Table A1. Comparisons of similarity of run and flight length distributions were performed using a two-sample Cramer–von Mises test. To find significant differences between populations, the Wilcoxon–Mann–Whitney test was used everywhere, except when comparing parameters of lysosomal tracks classified as co-moving with NP aggregates vs. the rest of them—in this case, we used a two-tailed paired Student’s *t*-test.

## 3. Results

### 3.1. Lysosomal Movements Are Characterized by a Heavy-Tailed, Lognormal Distribution of Run/Flight Lengths

We compared lysosomal movement trajectories in five cell types—noncancerous breast epithelial MCF-10A cells versus MDA-MB-231 and MCF-7 breast adenocarcinomas, and mouse embryonic fibroblasts (MEF) versus HT-1080 fibrosarcoma cells. Lysosomes were labeled with Lysotracker Red dye (Figure 1a) and imaged with a confocal microscope at a rate of 5.11 frames per second (fps) for periods of 3–5 min, collecting large data sets with *n* = 8–41 cells and 6623–19,215 lysosome trajectories per cell type. An example of typical lysosomal trajectories in MCF-10A cells is shown in Figure 1b (see Supplementary Movie S1). To quantify lysosome dynamics, we plotted lysosome mean square displacements (MSD) versus time, *t*, (MSD $\propto t^{\alpha }$) and computed exponents α and diffusion coefficients, *D*, for each cell type. MSD plots for all trajectories pooled from all cells of one type are shown in Figure 2a. A summary from analyzing each cell’s trajectories is shown in Figure 2b. As expected for lysosomes engaged in stop-and-go motion [16], the overall movements of lysosomes in all cell types—both cancer and noncancerous ones—were superdiffusive with exponent α ~ 1.29–1.36 (see Figure 2a,b, Table 1).

**Figure A1:**
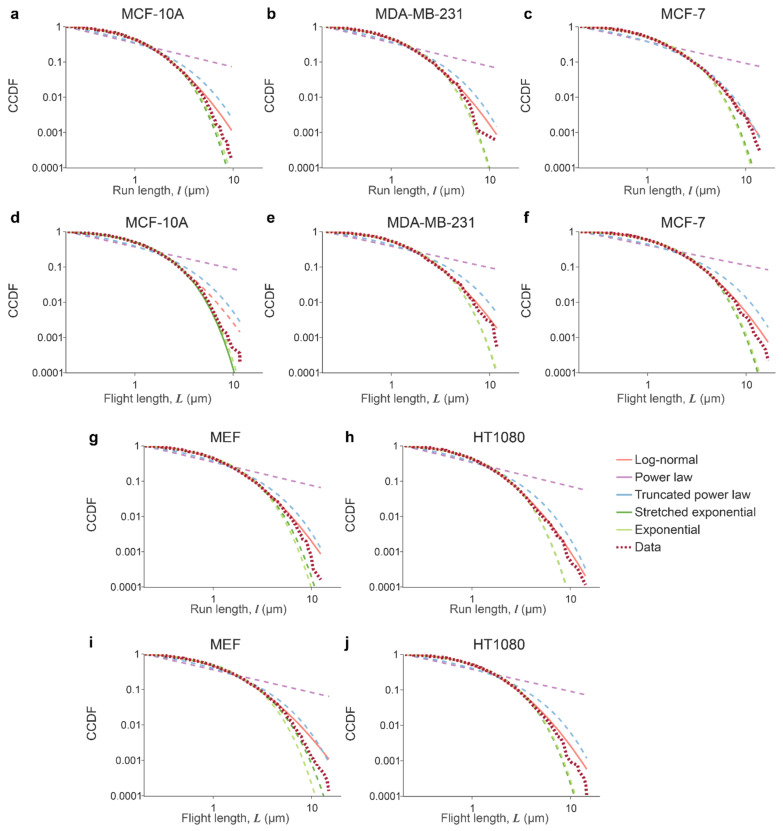
CCDFs of datasets with corresponding distribution fits. Subpanels show curves for run (**a**–**c**,**g**,**h**) and flight (**d**–**f**,**i**,**j**) lengths for MCF-10A (**a**,**d**), MDA-MB-231(**b**,**e**), MCF-7 (**c**,**f**), MEF (**g**,**i**), and HT1080 (**h**,**j**) cells. Data CCDF curves are in burgundy dotted lines; best-fit distributions are plotted in solid lines, while competing distributions are in dashed lines.

**Figure A2:**
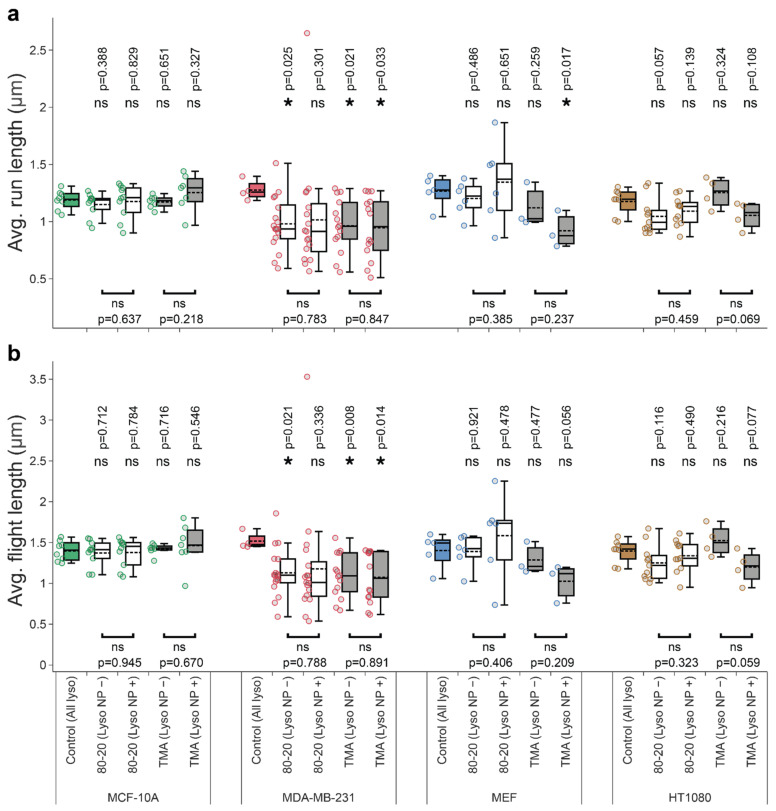
The effect of nanoparticle cargos on the average run (**a**) and flight (**b**) lengths. The notation of the box plots and experimental source data are identical to the main-text Figure 4 and Figure 5. Asterisk or ‘ns’ indicated in the lower part of the plots indicate presence or absence of statistically significant differences, respectively, between parameters for lysosomes with or without cargos determined with two-tailed paired Student’s *t* test (* *p* < 0.05). Similarly, asterisk or ‘ns’ above the box plots indicate statistical significance of the difference between lysosomes from cells treated with NPs (white and grey boxes) versus all lysosomes in untreated, control cells (colored boxes) determined using Wilcoxon–Mann–Whitney test (* *p* < 0.05). The analyses were based on the following number of cells and lysosome trajectories: MCF-10A/Control (*n* = 8 cells, *l* = 5260 lysosome trajectories); MCF-10A/80-20 (*n* = 10): NP− (*l* = 4177), NP+ (*l* = 597); MCF-10A/TMA (*n* = 12): NP− (*l* = 3769), NP+ (*l* = 303); MDA-MB-231/Control (*n* = 4, *l* = 1076); MDA-MB-231/80-20 (*n* = 17): NP− (*l* = 2771), NP+ (*l* = 260); MDA-MB-231/TMA (*n* = 14): NP− (*l* = 1618), NP+ (*l* = 116); MEF/Control (*n* = 5, *l* = 5781); MEF/80–20 (*n* = 6): NP− (*l* = 6272), NP+ (*l* = 131); MEF/TMA (*n* = 3): NP− (*l* = 5304), NP+ (*l* = 36); and HT-1080/Control (*n* = 8, *l* = 7953); HT/80–20 (*n* = 11): NP− (*l* = 4294), NP+ (*l* = 304); HT/TMA (*n* = 4): NP− (*l* = 2283), NP+ (*l* = 79).

To distinguish the active and passive movements, we computed Haar wavelet coefficients on the scale of 50 frames and assigned passive/active motion (as described in [35] and the Section 2). Trajectory segments with active motion were classified as “runs.” Consecutive “runs” in the same direction were grouped into “flights” (see Materials and Methods for details). We then proceeded to plot complementary cumulative distribution functions (CCDFs) of run/flight lengths (Figure 2e–h) and computed the percentage of time that lysosomes spent in active motion (Figure 2d and Figure A2, cf. next section). Plotting CCDFs on a log-log scale revealed that these distributions are heavy-tailed. Accordingly, we compared our experimental data to multiple competing heavy-tailed models using maximum likelihood estimates (MLE) and Akaike weights to determine the strongest supported model. The specific models compared were power law and truncated power law (both indicative of Lévy walks [21,22,24]), lognormal (observed previously in naïve T lymphocytes migrating in lymph nodes [25,51]), heavy-tailed stretched exponential [21], and exponential distribution (indicative of diffusive motions). All model comparisons are shown in Figure A1 in Appendix A and Table 1. These analyses revealed that lysosome movements in all cell types analyzed, except for MCF-10A, were characterized by heavy-tailed, lognormal distributions of run/flight lengths. For MCF-10A, the run length data fit lognormal distribution, while flight length data fit stretched exponential distribution better.

### 3.2. Tissue Origin and Cancer-Specific Differences in Lysosomal Dynamics

Inspection of the values of diffusion coefficients, *D* (Figure 2c, Table 1) and percentage of time spent in active transport (Figure 2d) revealed cell-type-specific differences in lysosomal dynamics. On one hand, the diffusion coefficients were higher for lysosomes in cells of epithelial origin (MCF-10A and MDA-MB-231, MCF-7) than those of fibroblast origin (MEF and HT-1080). Similarly, the former spent roughly twice as much time (on average 12, 15, and 17%, respectively) in active transport than the latter (7 and 9%) (Figure 2d). The percentage of steps detected in active motion is within the broad range of 5–30% reported for different vesicles by others [28,29,35]. On the other hand, the differences between noncancerous and cancer counterparts for the three metrics (α, *D*, % time in active transport) were not statistically significant (Figure 2a–d). However, closer inspection of CCDFs revealed differences in the distributions of run and flight lengths between cancer and noncancerous cell lines (Figure 2e–h). Here, the differences were cancer-specific—MDA-MB-231/MCF-7 cells’ lysosomes had slightly longer run/flight lengths than MCF-10A cells’ lysosomes. Conversely, MEF cells’ lysosomes had longer run/flight lengths than HT-1080 cells’ lysosomes (see Figure A2). The biological significance of these small differences is unclear. Nevertheless, these results illustrate the importance of cell line choices for studies designed to identify cancer-specific changes.

### 3.3. Wavelet-Based Approach for Detection of Lysosome-Nanoparticle Co-Movement

Next, we turned our attention to the co-movement of lysosomes with nanoparticle cargos. The first objective was to automatically extract the trajectories of lysosomes that carry NP cargos. Our CWT-based co-movement detection method recognized up to 27% of lysosomes inside NP-treated cells as cargo-bearing.

**Figure 3:**
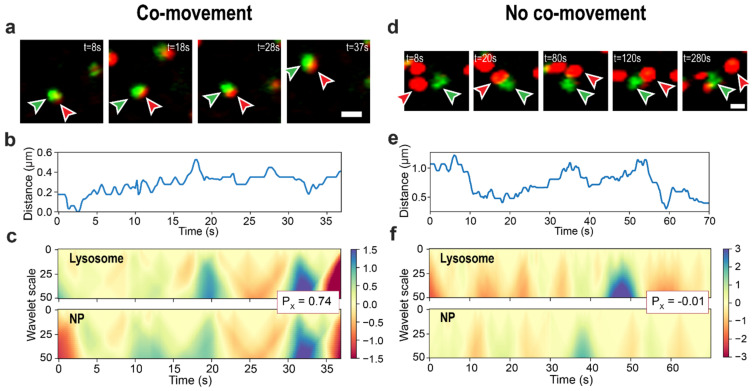
Examples of classification by the co-movement detection method. Lysosome–NP cluster pairs are correctly classified as moving together (**a**–**d**) and separately (**d**–**f**). (**a**,**d**) are lysosome (red) and NP-aggregate (green) experimental images prepared by combining images from the corresponding microscope channels. Arrows indicate the objects from the analyzed pair; the scale bars are 1 μm. (**b**,**e**) are center-to-center distances of the pairs. (**c**,**f**) demonstrate CWT coefficient maps for trajectories’ *x*-axes. Inset box with $P_{x}$ values show the Pearson’s correlation coefficient between lysosomal and NPs-aggregate CWT coefficient maps. For clarity, data only from the 0-70 s time interval (out of 300 s) are shown on (**e**,**f**). See Supplementary Movie S2.

Figure 1e shows images and trajectories of a typical lysosome-NP aggregate pair. In this and similar pairs, the lysosomes and the NP aggregates are transported together over the long time interval, with significant overlap between the fluorescence (Lysosome/red) and CRM channels (Au NP cluster, green; overlap shown in yellow), and are correctly classified by our method as co-moving. While this case is affirming, we present more challenging examples of the classification in Figure 3. The panels on the left (Figure 3) show a case of nanoparticle cargo transported together with a lysosome over a shorter distance and with minor overlap between the two channels. The panels to the right show a different example where particles have small average center-to-center distances and minor overlap between the two channels in selected frames but do not move together. Our algorithm classified both examples correctly—the first as co-moving and the second as not co-moving. In the first case, the average center-to-center distance (plotted in Figure 3b) was 0.3 μm, and in the second case, it was 0.8 μm, dropping to 0.3 μm at some points (Figure 3e). The distances are comparable, yet after inspecting the images for the second case (as shown in Figure 3d), it becomes apparent that the movements of the two particles are independent. This example shows that the center-to-center distances alone would not reject the false-positive co-movement detections—with a threshold too small, many object pairs with larger sizes would not be recognized as co-moving. Correlation coefficients between CWT data help clarify the situation—the co-moving pair has correlations of 0.74 and 0.97, while the independently moving pair has −0.01 and 0.07, for *x* and *y* axes, respectively (please refer to Figure 3c,f for the pairwise *x*-axis CWT coefficients maps), which leads to correct classification.

### 3.4. Mixed-Charge Gold Nanoparticles Selectively Disrupt Lysosomal Transport in Cancer Cells

**Figure 4:**
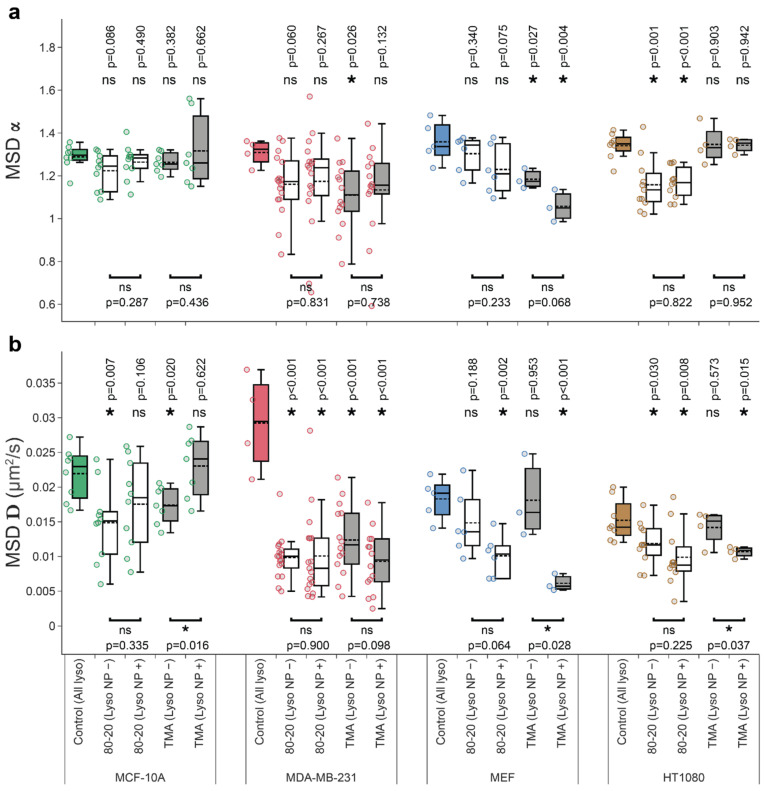
Cell type-specific effects of nanoparticle cargos on lysosome movement parameters. MCF-10A non-cancerous breast epithelial cells vs. MDA-MB-231 breast adenocarcinoma and normal mouse embryonic fibroblasts (MEF) vs. HT-1080 fibrosarcoma were untreated (Control), or treated with mixed-charge 80:20 or purely cationic TMA NPs. Trajectories for all lysosomes from each cell were pooled together to compute (**a**) exponent α and (**b**) diffusion coefficient *D*. For NP-treated samples, wavelet-based co-movement algorithm (see Materials and Methods) was used to further subdivide lysosomes into those carrying NP cargos (Lyso NP+) and those without detectable NP cargos (Lyso NP−). Data are displayed as box-and-whisker plots; boxes delineate the lower and upper quartiles of the data, middle lines show median values, dashed lines show mean vales, colored dots show lysosome dynamics parameters for each cell and whiskers show upper and lower extremes. Asterisk or ‘ns’ indicated in the lower part of the plots indicate presence or absence of statistically significant differences, respectively, between parameters for lysosomes with or without cargos determined with two-tailed paired Student’s *t* test (* *p* < 0.05). Similarly, asterisk or ‘ns’ above the box plots indicate statistical significance of the difference between lysosomes from cells treated with NPs (white and grey boxes) versus all lysosomes in untreated, control cells (colored boxes) determined using Wilcoxon–Mann–Whitney test. (* *p* < 0.05). The analyses were based on the following number of cells and lysosome trajectories: MCF-10A/Control (*n* = 8 cells, *l* = 5260 lysosome trajectories); MCF-10A/80-20 (*n* = 10): NP− (*l* = 4177), NP+ (*l* = 597); MCF-10A/TMA (*n* = 12): NP− (*l* = 3769), NP+ (*l* = 303); MDA-MB-231/Control (*n* = 4, *l* = 1076); MDA-MB-231/80-20 (*n* = 17): NP− (*l* = 2771), NP+ (*l* = 260); MDA-MB-231/TMA (*n* = 14): NP− (*l* = 1618), NP+ (*l* = 116); MEF/Control (*n* = 5, *l* = 5781); MEF/80–20 (*n* = 6): NP− (*l* = 6272), NP+ (*l* = 131); MEF/TMA (*n* = 3): NP− (*l* = 5304), NP+ (*l* = 36); and HT-1080/Control (*n* = 8, *l* = 7953); HT/80–20 (*n* = 11): NP− (*l* = 4294), NP+ (*l* = 304); HT/TMA (*n* = 4): NP− (*l* = 2283), NP+ (*l* = 79).

**Figure 5:**
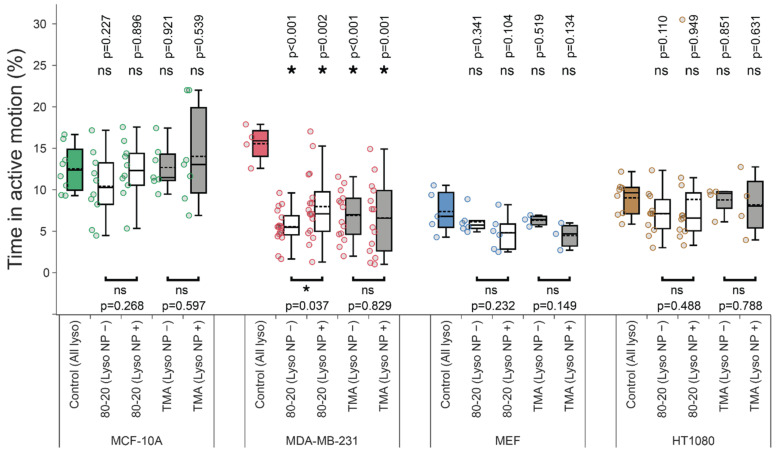
The effect of nanoparticle cargos on lysosome active transport. Time spent in active transport was computed after identifying active and passive trajectory segments with a wavelet-based approach. Note that for NP-treated samples, a wavelet-based co-movement algorithm (see Materials and Methods) was used to further subdivide lysosomes into those carrying NP cargos (Lyso NP+) and those without detectable cargos (Lyso NP−). Asterisk or ‘ns’ indicated in the lower part of the plots indicate presence or absence of statistically significant differences, respectively, between parameters for lysosomes with or without cargos determined with two-tailed paired Student’s *t* test (* *p* < 0.05). Similarly, asterisk or ‘ns’ above the box plots indicate statistical significance of the difference between lysosomes from cells treated with NPs (white and grey boxes) versus all lysosomes in untreated, control cells (colored boxes) determined using Wilcoxon–Mann–Whitney test. (* *p* < 0.05). All experimental details, statistical tests, and numbers of trajectories and cells analyzed are identical to Figure 4.

**Figure A3:**
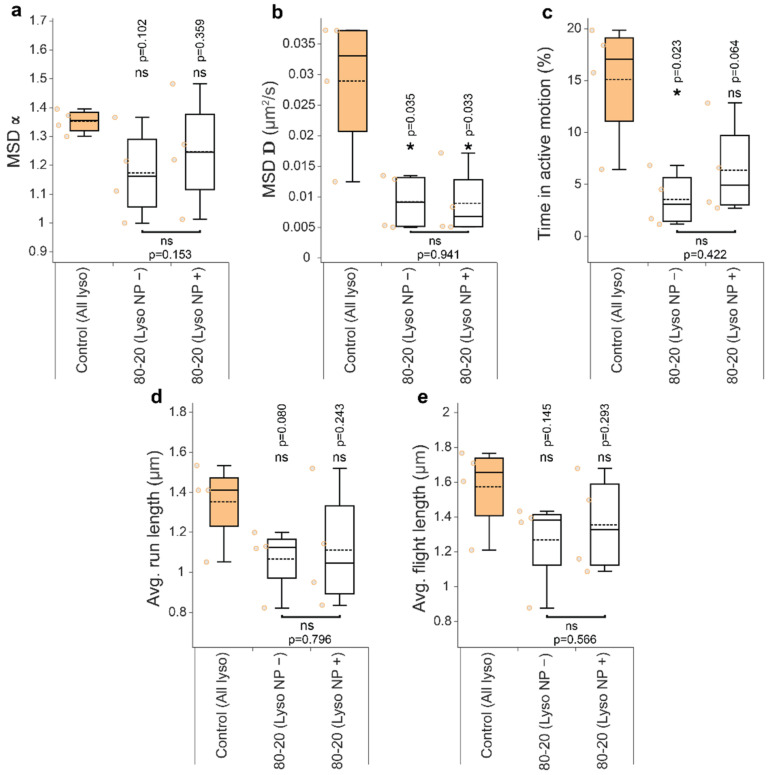
The effect of nanoparticle treatment on lysosomal motion parameters for MCF-7 cells. for all lysosomes from each cell were pooled together to compute (**a**) exponent α and (**b**) diffusion coefficient *D*, (**c**) percentage of time spent in active transport, and (**d**,**e**) average run and flight lengths. For more details, see Figure 4 caption. Asterisk or ‘ns’ indicated in the lower part of the plots indicate presence or absence of statistically significant differences, respectively, between parameters for lysosomes with or without cargos determined with two-tailed paired Student’s *t* test (* *p* < 0.05). Similarly, asterisk or ‘ns’ above the box plots indicate statistical significance of the difference between lysosomes from cells treated with NPs (white and grey boxes) versus all lysosomes in untreated, control cells (colored boxes) determined using Wilcoxon–Mann–Whitney test (* *p* < 0.05). Number of cells and trajectories used: Control (*n* = 4 cells, *l* = 1756 lysosome trajectories); 80–20 (*n* = 4): NP− (*l* = 369), NP+ (*l* = 31).

**Figure A4:**
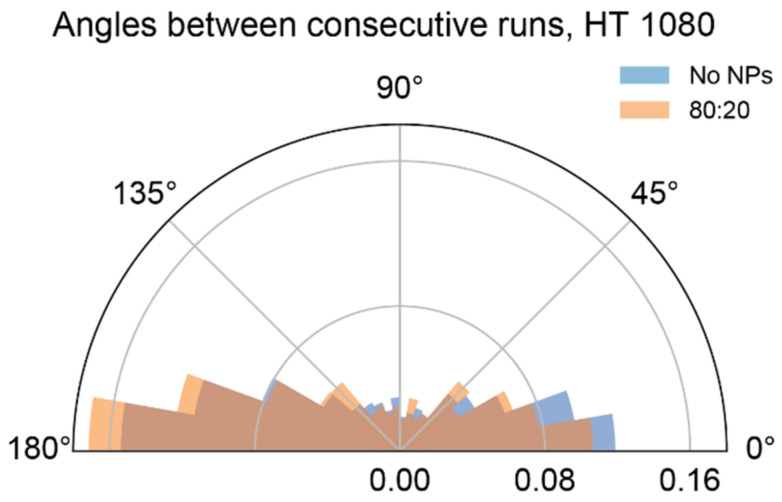
Distribution of turning angles between consecutive active runs in HT 1080 cells.

Mixed-charge nanoparticles selectively kill cancer cells through a lysosome-dependent mechanism [37]. We hypothesized that the crystallization of MCNPs in cancer lysosomes disrupted the transport of cargo-carrying lysosomes selectively in cancer cells. Here, we substantiated this hypothesis by quantifying various lysosome dynamics metrics in cancer and noncancerous cells exposed to 80:20 MCNPs. As a control, we also tested the effects of purely cationic TMA NP cargos, which were non-selectively toxic towards normal and cancer cells. With the method for detecting co-moving lysosome/NP pairs established in Section 3.3 above, we could use lysosome and NP trajectory data to automatically sort lysosomes in each cell into two groups—those carrying NP cargos and those without cargos—and analyze their trajectories separately. As predicted, we observed more pronounced inhibition of lysosome dynamics by mixed-charge 80:20 NPs in cancer cells than in normal cells (Figure 4, Figure 5 and Figure A2; data for MCF-7 are shown separately in Figure A3). The specific metrics affected by exposure to 80:20 NPs were cancer-specific—MDA-MB-231 lysosomes had lower diffusion coefficients, *D* (Figure 4) spent less time in active transport (Figure 5), and had smaller average run and flight lengths (Figure A2). Interestingly, in the case of HT 1080 cells with 80:20 NPs vs. no NPs, α and *D* values decreased (Figure 4), while the time spent in active motion and run/flight lengths (Figure 5 and Figure A2) did not change significantly. We found one possible explanation for that by considering angles between consecutive active runs (irrespective of what was happening between the runs or their belonging to active flights), as shown in Figure A4. In 80:20 NP treated cells, the proportion of runs in the same direction as a preceding run (around 0° in Figure A4) decreased, and the active motion in the backward direction (around 180°) became more prevalent than in untreated cells. This phenomenon could decrease the overall displacements from the starting point and, in turn, result in lower MSD parameters while not affecting the time spent in active transport.

When considering co-movements, typically, the effect of 80:20 NPs was general—that is, both lysosomes with cargos and those without detectable cargos were affected to a similar extent (Figure 4, compare ‘Lyso NP−‘ versus ‘Lyso NP+’ groups). Note that the ’Lyso NP−’ group may contain up to 20% of false-positives (lysosomes with cargos that escape detection, cf. Discussion), while the ‘Lyso NP+’ group did not contain any false-negatives. Yet another explanation—supported by TEM images and uptake of 50–100 nm-sized aggregates and their gradual coalescence into larger aggregates in cancer cells [37]—is that lysosomes in the ‘Lyso NP−‘ group contain smaller NP aggregates that are not detectable with confocal reflection microscopy. Still another possibility is that disrupted lysosomal transport results from the general effects of 80:20 NPs on cellular homeostasis (cf. Section 4).

Notably, lysosome transport disruption by 80:20 NPs occurred selectively in cancer cells. The effects of 80:20 NPs on the same metrics in noncancerous MCF-10A and MEF cells were marginal. One notable exception is the diffusion coefficient which was decreased for lysosomes without cargos and not affected in lysosomes with cargos for MCF-10A cells (Figure 4b). Conversely, *D* values for MEF cells’ lysosomes without cargos were unaffected, while lysosomes with NP cargos had lower diffusion coefficients (Figure 4b). These differences in diffusion coefficients certainly warrant a more detailed investigation of the heterogeneity of individual lysosomes/their trajectories in future studies. Importantly, however, the active transport in noncancerous cells was not inhibited by 80:20 NP cargos. Specifically, α exponent, % time spent in active transport, average run/flight lengths were similar in MCF-10A and MEF cells with or without 80:20 NP treatment (Figure 5 and Figure A2).

Consistent with their accumulation in lysosomes in MDA-MB-231 cells, purely cationic TMA NPs and 80:20 NPs had very similar effects of cancer lysosome dynamics. Unexpectedly, TMA NPs had more disruptive effects on lysosome transport in MEF cells than in HT-1080. Lysosome trajectory sorting using co-movement analysis (cf. above) revealed α<1, indicating subdiffusive, constrained motions and lower *D* values specifically for lysosomes with TMA NP cargos (Figure 4a,b).

Overall, these results are consistent with TMA NPs being indiscriminately toxic and 80:20 NPs being selectively toxic towards cancer cells as reported previously in [37].

## 4. Discussion

In light of the recent and dramatic shift in the understanding of lysosomes not merely as cell’s “recycle bins” but organelles with diverse functionality [1,2,3,6,52], the study of lysosomal motions and localization patterns appears timely and important. We discovered that the distribution of lysosomal transport lengths—both active runs and persistent flights—for the most part follows the heavy-tailed lognormal distribution (logarithm of which is normally distributed, hence the name). Such distributions are often found in nature and can result from multiplicative or branching random processes [53,54]. Lognormal distribution is often compared with another heavy-tailed distribution family—power law and its truncated version. In the context of movement patterns, power law-distributed steps indicate that the object moves according to a Lévy walk process [55] and might be following an optimal search strategy for finding rare targets [23]. In the context of organelles, Lévy walks have been used to describe endosomal transport [24]. Our findings that motions of lysosomes follow lognormal distribution point to a different model. Specifically, it has been shown that lognormal distribution might arise from a movement strategy that balances the number of total (repeated) target encounters with a wider search for new targets [25]. Given the variety and importance of contacts between lysosomes and other organelles [2], we see intriguing prospects in quantifying such interactions (for which our co-movement detection tool can be readily used) and painting a comprehensive picture of lysosomal behavior in the cell.

Changes in lysosomal sizes observed during cell differentiation [32], malignant transformation [7], or due to other perturbations [26] could affect lysosome dynamics parameters. Bandyopadhyay et al. showed that osmotic swelling of lysosomes lowers diffusive component, but does not affect the active transport of lysosomes in epithelial cells [26]. Durso et al. showed more complex dependencies in differentiating neuronal stem cells [32]. We did not observe similar correlations in untreated cells—lysosomes of similar average sizes could have *D* values differing two-fold (for example, HT-1080 vs. MDA-MB-231, see Appendix B, Table A2). Contrary to lysosomes swollen by sucrose treatment [26], larger lysosomes in MCF-7 had the highest *D* values. We did observe some decrease in *D* values for enlarged lysosomes due to the accumulation of 80:20 MCNPs (Figure 4b). Still, lower *D* values were also apparent in MCF-10A cells which internalized 80:20 NPs but did not have enlarged lysosomes, suggesting more complex regulation of lysosomal transport.

In the context of cytoskeleton elements underlying the active movement of lysosomes, the transport parameters could be affected by the density of microtubule (MT) intersections (providing roadblocks to continuous vesicle movement) and/or varying proportions of subclasses of MTs with different dynamic properties. The precise quantification of the former is challenging even with super resolution approaches due to the high density of the MT network and has only been studied in detail in selected epithelial cells [16,56]. However, it is particularly interesting that the MT assembly and disassembly rates are lower, and rescue frequencies are higher in epithelial cells than in fibroblast cells [57,58]. In other words, the MT network is more stable in epithelial cells. In addition, subpopulations of MTs with distinct dynamic properties have been detected in both epithelial cells [59] and fibroblast cells [58]. The meta-stable, longer-lasting MTs are typically characterized by the presence of various posttranslational modifications (PMTs) of α-tubulin, including detyrosination and acetylation of α-tubulin. For example, Mohan et al. showed that, in BS-C-1 epithelial cells, ~35% of MTs are detyrosinated, ~70% are acetylated, while ~50% of MTs contained both detyrosinated and acetylated α-tubulin [18]. Lysosomes and their target organelles were enriched on detyrosinated MTs in a manner dependent on motor protein kinesin-1. Furthermore, the movements of lysosomes on such detyrosinated MTs were impaired (resulting in shorter run lengths), facilitating lysosome contacts with target organelles [18]. In a different study, Hao et al. showed that Golgi-associated MTs had fewer intersections with other MTs, were stabilized with limited acetylation and detyrosination contributions, and provided stable tracks for fast polarized cargo transport to the leading edge of migrating epithelial cells [56]. It would be interesting to examine if higher *D* values and more time spent in active transport for cells of epithelial origin observed in our experiments were due to lysosome transport along specific subsets of such stabilized MT tracks and if they rely on specific kinesin motor proteins [60]. It would also be interesting to study if NP cargos alter the type of MT tracks (and motor proteins) used for lysosome transport.

In the research concerning cargo transport and co-transport on the level of individual organelles, the most widespread techniques to detect such events from microscopy images are manual detection [61], pointwise trajectory correlation [62], and colocalization-based [45,46,63,64] methods. However, the manual approach is not feasible for large-scale studies such as ours—simple pointwise correlation might not be representative enough since it does not consider the scale of movements, while the colocalization is based on the assumption that there is an overlap between the objects in images. In our experiments, fluorescence quenching by Au NPs aggregates [65] interferes with the Lysotracker dye intensity. In addition, lysosomes and NP aggregates were occasionally observed to move together in the absence of overlap between fluorescence (for lysosomes) and reflection (for NP aggregates) channels (see example in Figure 3a). The latter corresponds to lysosome-autolysosome pairs in TEM images (shown in Figure 6 in [37]) engaging in limited fusion interactions of the “kiss-and-run” type. A colocalization-based approach would miss such interactions. Moreover, one of the main goals of our present study was to find lysosomes that carry cargo in a crowded cellular environment, where spurious colocalization may arise without co-transport, e.g., when one object is below another. For these reasons, we ruled out colocalization as a metric in our study—that, and the fact that the center-to-center distance alone was not sufficient to reliably isolate cargo-carrying lysosomes, prompted the development of our co-movement detection method.

Wavelet transform-based analysis is a widely employed tool across all scientific and engineering disciplines dealing with time-series data [66,67,68,69], with successful examples in the analysis of endosomal [24] and lysosomal motion [36]. Since CWT is essentially a convolution of wavelet kernel taken with multiple sizes, the resulting coefficients represent features on multiple scales. In the case of time-series data, scales represent frequencies, and CWT preserves time information about the amplitude of these frequencies in time (in contrast to Fourier transform, which gives information about the amplitude of certain frequencies, but not about the time of its occurrence). This makes wavelets instrumental in detecting correlation patterns in the data [70,71], specifically in tracking and co-movement analysis [72,73,74]. For our data, arguments can be made that both high- and low-frequency motions can indicate whether the objects are moving together or not: Brownian motion and fast individual steps of active movement appear at higher frequencies, while long-range transport and drift of object pairs belong to the lower frequency range. We found that Pearson’s correlation between CWT coefficients on multiple scales is an effective measure since all frequencies are taken into account, and correlations of multiple motion modes affect the classification results.

After a manual survey of our classification results, we could not identify false-positive detections of co-moving pairs; the number of false-negatives (that is, pairs that appeared to be moving together but were not classified as such) varied between populations, but was typically below ~20%, which was satisfactory for our goal of isolating the certainly co-moving lysosome–NP aggregate pairs. Predictably, lower correlation and higher distance thresholds decreased the number of false-negatives but at the cost of occasional false-positive detections—hence, we set the thresholds at higher values to keep the subpopulation of co-moving pairs as “clean” as possible. This way, our imaging and data processing protocols allowed the extraction and analysis of bona fide parameters for cargo-bearing lysosomes from the rest of the tracks, and comparison with their counterparts, which represent mostly (though not exclusively) lysosomes without cargos (cf. Discussion below).

The presence of some cargo-bearing lysosomes in Lyso NP− group (cf. Discussion above) may partly explain similar effects of 80:20 NPs on lysosome dynamics parameters in Lyso NP− and Lyso NP+ groups (cf. Section 3.4 and Figure 4 and Figure 5). A more likely explanation, however—supported by TEM images and uptake of 50–100 nm-sized aggregates and their gradual coalescence into larger aggregates in cancer cells [37]—is that lysosomes in ‘Lyso NP−‘ group contain smaller NP aggregates that are not detectable with confocal reflection microscopy. More sensitive detection of NP cargos via superresolution reflectance imaging [75] or dark-field microscopy [76] could help resolve these questions. It is also conceivable that 80:20 NPs could affect all cancer lysosomes through general perturbations of cells’ homeostasis, such as nutrient deprivation through inhibitions of lysosomal degradative capacity. Cell starvation is known to decrease cytoplasmic pH, which induces juxtanuclear accumulation of lysosomes where they are more likely to come in contact and fuse with autophagosomes [77]—in this scenario, lysosome motions would be constrained. As mentioned above, however, the Lyso NP+ group identified with co-movement algorithm contains a pure population of lysosomes with NP cargos, making this data exceptionally reliable.

## 5. Conclusions

In summary, we devised an algorithmic workflow with which to extract and quantify the motion patterns of cellular organelles in addition to those of organelles carrying particulate cargos. Here, we applied this workflow to study a large body of lysosomal trajectories, confirming superdiffusivity of lysosomes’ motions but also discovering the previously unreported lognormal distribution characterizing lysosomal transport lengths. In this context, the hypothesized connection between this lognormal distribution and lysosomes’ “patrolling” strategy (possibly balancing the number of repeated encounters with the efficiency of search for new targets) appears exciting and certainly merits additional research and verification. In the meantime, we hope that the codes we make available will stimulate similar analyses of additional cell types and organelles other than lysosomes.

## Figures and Tables

**Table 1:** Statistical analysis of lysosome movements in cancer and noncancerous cells. The exponent *α* and diffusion coefficient *D* values were computed from mean square displacement (MSD) versus time plots shown in Figure 2a. Data = mean ± s.d. *n* = 4–8 cells (see below). The fit parameters for run and flight lengths—from lognormal (or stretched exponential where appropriate) distributions—and Akaike weights for all model comparisons are shown (see Section 2 for details). LN = lognormal, P = power law, TP = truncated power law, SE = stretched exponential, E = exponential. The strongest supported model is indicated in bold. Complementary cumulative distribution functions (CCDFs) for run (*l*) and flight (*L*) lengths shown in Figure 2e–h for all cell types fit lognormal distributions, except MCF-10A for which the CCDF for *L* fits stretched exponential distribution. See also Figure A1 for model fits. The number of cells and lysosome trajectories analyzed were as follows: MCF-10A (*n* = 8 cells, *l* = 5260 lysosome trajectories), MDA-MB-231 (*n* = 4, *l* = 1076), MCF-7 (*n* = 4, *l* = 1756), MEF (*n* = 5, *l* = 5781), and HT-1080 (*n* = 8, *l* = 7953).

Cell Type	MSD (*a*)	D (μm^2^/s)	Fit Parameters	Akaike Weights				
				LN	P	TP	SE	E
MCF-10A	1.29 ± 0.05	0.022 ± 0.004	*Runs*: μ = −0.193; σ = 0.804 *Flights*: λ = 0.840; β = 1.037	**1** 0	0 <0.01	<0.01 <0.01	<0.01 **0.83**	<0.01 0.17
MDA-MB-231	1.31 ± 0.05	0.029 ± 0.006	*Runs*: μ = −0.147; σ = 0.833 *Flights*: μ = 0.028; σ = 0.838	**0.99** **1**	<0.01 <0.01	<0.01 <0.01	<0.01 <0.01	<0.01 <0.01
MCF-7	1.35 ± 0.04	0.029 ± 0.010	*Runs*: μ = −0.013; σ = 0.834 *Flights*: μ = 0.151; σ = 0.838	**1** **1**	0 0	0 <0.01	<0.01 <0.01	<0.01 <0.01
MEF	1.36 ± 0.09	0.018 ± 0.003	*Runs*: μ = −0.175; σ = 0.856 *Flights*: μ = −0.155; σ = 0.929	**1** **0.99**	0 0	<0.01 <0.01	<0.01 <0.01	<0.01 <0.01
HT-1080	1.34 ± 0.06	0.015 ± 0.003	*Runs*: μ = −0.183; σ = 0.802 *Flights*: μ = −0.019; σ = 0.829	**1** **1**	0 0	0 0	<0.01 <0.01	<0.01 <0.01

## Data Availability

The custom-written integrated workflow code that includes (i) detection of active movement from 2D trajectories by using continuous wavelet transform (CWT) approach; (ii) detection of co-movement of two types of particles/vesicles by using CWT; (iii) construction of complementary cumulative distribution functions (CCDFs) of run/flight length and model fitting, is available at https://github.com/conspol/cwt-active-comovement accessed on 10 January 2022.

## Supplementary Materials

The following supporting information can be downloaded at https://www.mdpi.com/article/10.3390/cells11020270/s1: Movie S1—Lysosome trajectories in MCF-10A cell. An overlay of differential interference contrast (DIC) channel showing cell outline, Lysotracker Red channel showing lysosomes, and lysosomal trajectories of an example MCF-10A cell in the absence of nanoparticle treatment. Time, h:m:s. Scale bar, 10 μm. See more examples in Supplementary material of [37]; Movie S2—Lysosome and nanoparticle aggregate co-movement analysis. An overlay of fluorescence (Lysotracker Red = lysosomes, red) and reflection (Au NPs, green) channels in MCF-10A cell treated with 80:20 NPs; example region from a single cell is shown. Time, h:m:s. Scale bar, 2 μm. See more examples in Supplementary material of [37].

## Appendix B

**Table A1:** Distributions used in model comparison. Here, x is observed data, a is the minimal data point, Γ is upper incomplete gamma function, erfc is complementary error function, and μ, λ, σ, β are parameters of the corresponding distributions.

Distribution Name	Probability Density Function p(*x*)
Power law	$\frac{x^{-\mu }}{a^{1-\mu }\;}\left(\mu -1\right)$
Truncated power law	$x^{-\mu }e^{-\lambda x}\frac{\lambda^{1-\mu }}{\Gamma \left(1-\mu ,\;\lambda a\right)}$
Log-normal	$\frac{1}{x}\exp\left[-\frac{{\left(\ln x-\mu \right)}^{2}}{2\sigma^{2}}\right]\sqrt{\frac{2}{\pi \sigma^{2}}}{\left[\mathrm{erfc}(\frac{a-\mu }{\sqrt{2}\sigma })\right]}^{-1}$
Stretched exponential	$\beta \lambda x^{\beta -1}e^{\lambda \left(a^{\beta }-x^{\beta }\right)}$
Exponential	$\lambda e^{\lambda \left(a-x\right)}$

**Table A2:** Lysosomal diameters in all cell types. Lysosome sizes in all cell types analyzed in the present study without or with 80:20 NP treatment were quantified from confocal midplane images of Lysotracker-stained organelles. Data = mean ± s.d. A two-tailed Student’s t-test with unequal variances between control and NP-treated groups. * *p* < 0.05; ^ns^ = not significant. Data are from Borkowska et al., 2020 [37].

Cell Type/Treatment	Lysosomal Diameter [μm]	Lysosomes, *l*	Cells, *n*
MEF	0.65 ± 0.23	525	7
MEF + 80:20 NPs	0.71 ± 0.25 *	759	6
HT-1080	0.67 ± 0.24	707	6
HT-1080 + 80:20 NPs	0.99 ± 0.34 *	539	11
MCF-10A	0.54 ± 0.20	316	10
MCF-10A + 80:20 NPs	0.57 ± 0.22 ^ns^	340	12
MDA-MB-231	0.67 ± 0.30	418	16
MDA-MB-231 + 80:20 NPs	1.00 ± 0.49 *	235	10
MCF-7	0.80 ± 0.35	250	11
MCF-7 + 80:20 NPs	1.42 ± 0.92 *	239	11
